# Anticancer properties of chitosan against osteosarcoma, breast cancer and cervical cancer cell lines

**DOI:** 10.22088/cjim.10.4.439

**Published:** 2019

**Authors:** Zeinab Abedian, Ali Akbar Moghadamnia, Ebrahim Zabihi, Roghayeh Pourbagher, Masoumeh Ghasemi, Hamid Reza Nouri, Hamed Tashakorian, Niloofar Jenabian

**Affiliations:** 1 Student Research Committee, Babol University of Medical Sciences, Babol, Iran; 2 Dental Materials Research Center, Health Research Institute, Babol University of Medical Sciences, Babol, Iran; 3 Cellular and Molecular Biology Research Center, Health Research Institute, Babol University of Medical Sciences, Babol, Iran; 4 Department of Pharmacology, Babol University of Medical Sciences, Babol, Iran

**Keywords:** Apoptosis, Cell line, Chitosan, Cytotoxicity, Molecular weight, Necrosis.

## Abstract

**Background:**

Cancer is still the most common cause of morbidity in the world. Chitosan, a commonly used natural polymer, is consisted of different molecular weight with different biological activities.The purpose of this study was to determine cytotoxicity effect of high molecular weight (HMWC) and low molecular weight of chitosan (LMWC) on three cancerous cell lines MCF-7, HeLa and Saos-2 with different histological origin.

**Methods:**

The anticancer property of two types of chitosan on three cancerous cell lines and human fibroblast as normal cell line, was evaluated by cytotoxic activity including their apoptosis induction properties. Chitosan solutions 2% (w/v) were prepared. The cells were treated by different concentration of chitosan and viability was determined by MTT assay after 24, 48 and 72 h .Also the mode of cell death-apoptosis vs necrosis ,was determined by Annexin V staining assay and analyzed by flow cytometry.

**Results:**

While both types of chitosan were effective in inhibiting cell proliferation of three cancerous cell lines, fibroblast cells showed somehow more compatibility with chitosan. Despite of a significant decrease in all 3 cell lines viability, up to 90%, but we didn't see a concentration dependent difference between both types of chitosan (HMWC and LMWC) in their cytotoxic effects. Flow cytometry analysis showed necrosis more observable with MCF7 while the apoptosis pattern of death was more in Saos-2 and HeLa. Also, higher viability with both types of chitosan was seen in fibroblast as normal cells.

**Conclusion:**

While chitosan is compatible with normal diploid fibroblast cells, it shows anticancerous effect against 3 cancerous cell lines. Furthermore, it seems that the molecular weight of chitosan does not affect its anticancerous property.

Cancer is still a major public health problem and is one of the most common cause of morbidity globally. Shall be inhibited by new anticancer candidates, cell proliferation is one of the major steps in cancer cells metastasis of a multistep process that includes cell adhesion, invasion, cell proliferation, transport through circulatory system and growth in a secondary organ (1, 2). As the consequence of serious adverse reactions of chemotherapy, the main purpose of this research is to investigate new anticancer candidate with potential low cytotoxicity against normal tissues. Chitosan is one of the natural substances that has been used considerably for biomedical applications due to its high biocompatibility, biodegradability, low toxicity and intrinsic antibacterial activity (3). Owing these properties, chitosan has become excellent candidate for developing new anticancer agent. Chitosan is a polysaccharide comprising copolymers of glucoseamine and N-acetylglucosamine (4, 5) and can be derived by partial deacetylation of chitin which is naturally present in crab, shrimp, shells, lobster, coral, jellyfish, butterfly, ladybug, mushroom, and fungi (6, 7). 

There are many reports about antitumor activity of chitosan in which, its mechanism is related to membrane-disrupting and apoptosis-inducing activities (8). The anticancer properties of chitosan have been postulated by its antiproliferative action against human monocytic leukemia cell line (THP1) (9) as well as some human breast cancer cell lines (10). Necrotic effect was reported in human gastric carcinoma cell line MGC803 (11, 12). Qi et al. worked on antitumor effect of chitosan on hepatocellular carcinoma and showed that antitumor mechanism was mediated by the decrease of mitochondrial membrane potential, induction of membrane lipid peroxidation and neutralization of cell surface charge (13). Takimoto et al. showed that chitosan induced apoptosis by modulating death receptor expression and activating caspase-8(14). But Hasegawa et al reported that chitosan induced apoptotic death of bladder tumor cells via caspase-3 activation (15).

We would like to investigate about anticancer activity of the (LMW 100-300 kDa) and HMW 600-800 kDa) chitosan using human breast cancer cell line (MCF-7), human epithelial cervical cancer (HeLa), osteosarcoma (Saos-2) and noncancer fibroblast. Their cytotoxic activity was studied by MTT assay, and anticancer activity of apoptosis and necrosis were analyzed by flow cytometry. We therefore performed the present research to explore the anticancer effects of LMWC on cancerous cell lines in comparison with HMWC in terms of cytoxicity and apoptosis.

## Methods

***Materials: ***The following chemicals and reagents were obtained from the companies mentioned below. Chitosan, with two different MW: 600-800 kDa and 100-300 kDa with deacetylation degree of 75%-85% were purchased (ACROS, USA). RPMI 1640 (Gibco, Germany) Trypsin–EDTA (Sigma-aldrich), MTT (3-(4, 5-Dimethyl thiazol-2-yl)-2, 5-Diphenyl tetrazoliumbromide (ACROS, USA), FBS (Gibco,), Penicillin/Streptomycin (PAA), DMSO (Sigma-aldrich,), ApoFlowExFITC kit Exbio (ED7044).

***Cell-Lines: ***Three different human cell lines: MCF-7 human breast cancer), HeLa (human epithelial cervical cancer), Saos-2 (osteosarcoma) were purchased from the Pasteur Institute of Iran. Cancer cells were grown and maintained in Roswell Park Memorial Institute medium (RPMI) 1640 supplemented with 1% penicillin-streptomycin, 10% (v/v) heat-inactivated fetal bovine serum. The cells were maintained at 37º C under 5% CO2. The human fibroblast cell line was isolated from normal children’s foreskin by non- enzymatic method which had been cryopreserved since previous project with Ethical code: MUBABOL.REC.1390.8. (16). All cell lines were used to evaluate the effects of HMWC and LMWC in vitro. 

***Preparation of chitosan solution: ***LMW and HMW chitosan were dispersed in a 1% (v/v) glacial acetic acid (Merck, Germany) for the preparation of chitosan solutions 2% (w/v) and kept stirring at 50  C overnight for complete dissolution of their particles. Then the solutions were autoclaved at 121 C for 15 min and stored at 4 C until used (17-20).

***Cell cytotoxicity*** ***assay: ***Cell viability assay was carried out using the MTT (3-(4, 5-dimethylthiazol-2-yl)-2,5-diphenyltetrazolium bromide),which measures the activity of mitochondrial dehydrogenase in the cells. For cell treatment, 5*10^3^ cells were seeded into each well of a 96-well flat bottom plate (Orange scientific) and the plate was incubated at 37°C in a humidified CO_2 _5%. After 24 h, the cell culture medium was replaced by a fresh medium that contained HMW and LMW chitosan at final concentrations of 0.25, 0.5,1,2,4 mg/mL separately. After 24, 48 and 72 h MTT solution (5 mg/mL) was freshly prepared and added to each well. Three hours after incubation, the formed formazan crystals were dissolved in DMSO and the resulting color was measured spectrophotometrically using a microplate reader (Rayto, China) at a test wavelength of 570 nm and a reference wavelength of 630 nm. Each experiment was performed in triplicate. Same cell lines without exposure of both types of chitosan served as controls. Percentage cell viability was calculated by dividing the absorbance of individual wells by the mean absorbance of control wells (21, 22).

***Apoptosis by flow cytometry: ***To determine whether the initial cell death occurred in MCF-7, HeLa, Saos-2, and skin fibroblast exposed LMW and HMW could be due to apoptosis (a programmed cell death), annexin V/PI test was carried out. Annexin V is intracellular protein that binds to phosphatidylserine (PS). PS is normally only found on the intracellular leaflet of the plasma membrane in healthy cells but during early apoptosis, membrane is removed and PS translocates from inner phospholipid layer to the cell surface. Fluorochrome-labeled annexin V can identify apoptotic cells and for distinguishing between the necrotic and apoptotic cells propidium iodide solution (PI) was used which is a fluorescent dye that binds to DNA. The flow cytometry test can differentiate early apoptotic cells (FITC+/PI-), late apoptotic cells (FITC-/PI+),necrotic cells (FITC+/PI+) and intact cells (FITC-/PI-)(23,24). Annexin V/PI test was carried out according to protocol. Briefly, the treated cells were harvested by centrifugation and supernatant was removed. Washing of pellet was done with cold PBS. Cells were resuspended in 100 μL 1 x annexin V binding buffer, then.5 μL annexin V and 5 μL PI was added to cell suspension. Tubes were incubated in the dark for 15 minutes at room temperature. After centrifugation, cells were resuspended in 100 μL annexin V binding buffer then were analyzed by flow cytometer (PARTEC, Germany).

***Statistical analysis: ***Statistical analysis was performed using SPSS Version 19. The t-test and analysis of variance (ANOVA) via Tukey's multiple comparison were used to determine the different variable effects on cell lines. The difference between numerical results was considered in a significant level in p*<*0.05.

## Results

**Figure 1:**
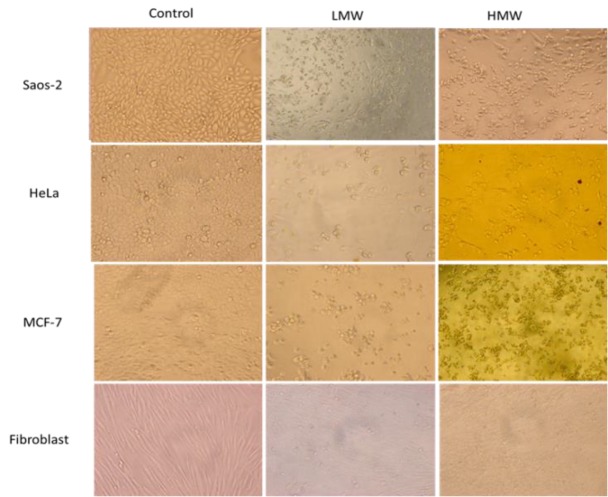
Inverted microscopy micrographs of untreated cells as control and treated cells with 1mg/mL HMW and LMW after 48h .Control cells demonstrated dense cell populations, against treated cells. (X 20)

***Cell Cytotoxicity assessment: ***Three cancer cell lines MCF-7, HeLa, Saos-2 and fibroblast isolated from foreskin were treated with a different concentration, (0, 0.25, 0.5, 1, 2, 4 mg/mL) of LMW and HMW chitosan and cytotoxity was measured after 24, 48 and 72 h incubation by MTT assay. The morphologic changes were seen in fig 1.

**Figure 2:**
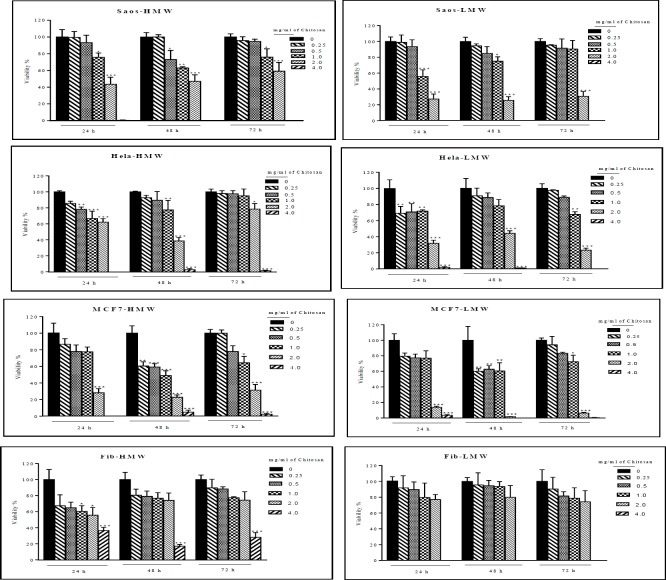
Effect of exposure high and low molecular weight chitosan with different concentrations on the viability of MCF7, Saos-2, HeLa as cancer cell lines and fibroblast derived from foreskin as non-cancer cell. Apart from fibroblast, the results of MTT assay, for each cancer cell lines showed significant differences compare to control (0 concentation of chitosan) (* P<0.05 vs control, **P<0.01 vs control, ***P<0.001 vs control). Data represent mean±SEM and experiments performed in triplicate. ns: no significant, HMW: High molecular weight, LMW: Low molecular weight

As shown in figure 2, cytotoxic activity of both types of chitosan was concentration-dependent but varied among cells tested. With increasing concenteration of HMW and LMW chitosan, inhibition rate increased and viability of cells significantly decreased. Both types of chitosan were more efficient in inhibiting cell proliferation of three cancer cell lines than fibroblast as non-cancer cell and cell viability reduced concentration-dependently up to 70-90% of the control with chitosan to 2 mg/mL in cancer cells (p<0.05). The effect of HMW chitosan on Saos and HeLa was more than LMW of chitosan because the concentration of 0.5 mg/mL of HMW chitosan significantly decreased viability of cells but LMW chitosan was efficient in 1mg/mL. IC50 value for HMW chitosan in MCF7, HeLa, and Saos-2 was 1.68, 1, 1.7, mg/mL and for LMW was 1.76, 1 and 1.63 mg/mL, respectively. 

Comparision of cytotoxicity between cancer cells and fibroblast derived from foreskin as non-cancer cells showed that the viability of dermal fibroblast was higher than cancer cells after exposure to HMW and LMW chitosans and both types of chitosan have less cytotoxicity on dermal fibroblast than cancer cell lines, it means that their side effects are low.

**Figure 3:**
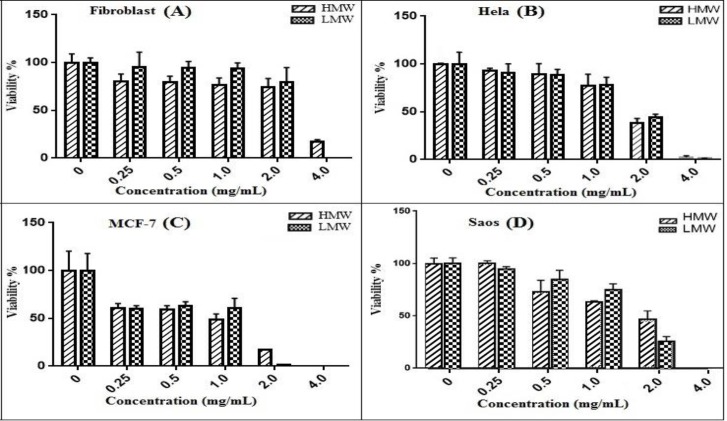
Comparison of HMW and LMW chitosan on fibroblast (A), HeLa (B), MCF-7 (C) and Saos-2(D), after 48 h treatment shows that there is no significant difference between them (P>0.05) except for 4mg/mL on fibroblast and 2 mg/mL on MCF-7(P<0.01).The results are the means±SEM and experiments performed in triplicate

The effect of HMW and LMW chitosan on all types of cell lines was compared to each other and there was no significant difference between HMW and LMW chitosan (p>0.05) (fig 3).

***Quantification of apoptosis by flow cytometry: ***Annexin V/PI test was carried out for the determination of mode of cell death after exposure to 1 and 2 mg/mL of HMW and LMW chitosan for 48 h. Apoptotic and necrotic incidence in MCF7, Saos-2, HeLa as cancer cell lines and dermal fibroblast as normal cell were determined by flow cytometry and overall obtained result confirmed the presence of apoptosis and necrosis in chitosan-treated cancer cells. The apoptotic incidence increased to 34% and 20.46% in Saos-2, 7.75% and 5.9% in MCF7, 63% and 44.68% in HeLa after being treated with 1mg/mL HMW and LMW chitosan, respectively. 

**Figure 4:**
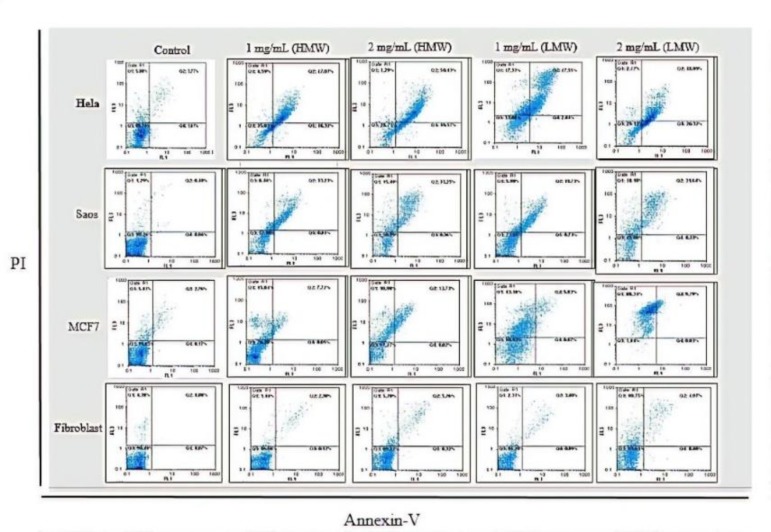
Determination of the mode of HeLa, Saos-2, MCF7 and fibroblast death using Annexin/ PI staining and flowcytometry. The diagram shows the status of the cells after exposure to the two concentrations (1 and 2 mg/mL) of HMW and LMW chitosan and implies that chitosan can induce both apoptosis and necrosis in cancer cell lines but fibroblast as normal cells was intact

The necrotic incidence was 8.46% and 5.98% in Saos-2, 15.84% and 43.16% in MCF7, 2% and 1.27% in HeLa after being treated with 1mg/mL HMW and LMW chitosan, respectively. In contrast, fibroblast treated with 1 mg/mL HMW and LMW chitosan were like untreated cells and 94% to 96% of cells were intact (fig 4).

## Discussion

In this study, chitosan with high (600-800 kDa) and low (100-300 kDa) molecular weights were used to compare their effects on three cancer cell lines, including MCF-7, HeLa, Saos-2 and fibroblast derived foreskin as normal cell. The results of MTT assay in the present study, demonstrated that HMW and LMW chitosan could exert cytotoxicity against MCF-7, Saos-2, and HeLa cell lines and both types of chitosan have less cytotoxic properties on dermal fibroblast, as a normal cell, than cancerous cell lines. Exposure of three cancer cell lines to chitosan showed that there is a concentration-dependent decrease in cell biomass(with IC50 value from 1 mg/mL up to 1.76 mg/mL. after 48 h of treatment)concentration-dependent (fig 2).

According to the results, there is no significant difference between the cytotoxic effects of high and low molecular weight chitosan on three cancerous and normal fibroblast cell lines except for the concentration of 2 mg/mL on MCF-7 and 4 mg/mL on fibroblast (p<0.01) (fig 3). In contrast to our findings, in the previous studies conducted on chitosan with different MW, there was a size dependent difference in cytotoxicity. However, the chitosan samples used in those studies were (chitosan carboxymethyl derivatives (CMCS), chitosan thymine conjugate, sulfated chitosan (SCS), sulfated benzaldehyde chitosan (SBCS), glycol-chitosan (GChi), N-succinyl chitosan (Suc-Chi) conjugates, furanoallocolchicinoid chitosan conjugate, Chitosan–metal complex and polypyrrole chitosan (25) derivatives of chitosan and also their MW were much lower than our samples. It also might indicate that from a special range of molecular weight, the cytotoxic effects of chitosan are not affected by its molecular size and there is no difference.

The results of flow cytometry analysis implied that the action mechanism of chitosan in the three cancer cell lines might be different. While apoptosis was the most obvious mode of death in HeLa and Saos-2 cell lines exposed to chitosan, necrosis was prevalent in the MCF-7 cell line. It seems that chitosan with HMW and LMW induces death in each cancer cell line. This finding was also observed with both different concentrations of chitosan (Fig 4).

In a previous study, short-term LMWC exposure, was less cytotoxic to HaCaT cells, as normal keratinocyte cells, than to Ca9-22 cells as oral squamous cell carcinoma. The anticancer activity was exerted through induction of apoptosis suggesting that LMWC could be a promising natural anticancer agent with fewer side effects on normal cells (26).

The effect of chitosan-copper complex on HeLa and 293 cell line showed that this complex inhibited tumor cell proliferation at 10^3 ^mol/L concentration, but not the normal human lung fibroblast cell line HLF, similar to our study.

As a consequence of difference in mechanism of cytotoxicity, there are contrary effects of chitosan on the cancer cell lines and fibroblast. Cancer cells have greater negative charge than normal cells which can be attracted more by positive charged amino groups of chitosan (27). Chitosan might disrupt the membrane cell through electrostatic interaction, leading to secretion of inflammatory cytokines, i.e. IL-6 and IL-8. These cytokines are mitogen for normal fibroblast but cancer cells might lose their ability to respond to the membrane-damaging effects of chitosan, including the release of inflammatory cytokines. Chitosan might directly attack cancer cells through interaction with the tumor cell membrane or extracellularly via a specific receptor, or via endocytosis (28, 29). 

Based on Ming Jiang is study, it was found that 24-h exposure of MCF-7 cells to 50 μM chitosan derivatives such as sulfated chitosan (SCS) and the sulfated benzaldehyde chitosan (SBCS) significantly inhibited cell proliferation and induced apoptosis. SBCS had better inhibitory effects and a lower IC50 compared to SCS, but our study showed necrosis was prevalent in the MCF-7 cell line ,it may be the chitosan exposure, was 48 h and also only chitosan was used ,not chitosan compound (10).

Antitumor effects of various low molecular weight chitosans (21-kDa, 46-kDa, and 120-kDa) and HMW chitosan (average 650 kDa), in sarcoma 180–bearing mice showed that chitosan with molecular weights of 21 kDa and 46 Da had antitumor activity while this effect was not observed in the mice treated with 130-kDa and 650-kDa chitosan (30).

Moreover human gastric carcinoma MGC803 cell line was sensitive to chitosan nanoparticles with an IC50 value of 3 µg/mL after 48 h treatment (11). The biological effect of chitosan on three human melanoma cell lines (not three cancer cell lines) was distinctive, the adhesion of primary melanoma A375 cell line and proliferation of primary melanoma SKMEL28 cell line decreased and induced apoptosis in RPMI7951 cell line similar to our study findings (31).

The HMWC cytotoxicity test using MTT assay against HepG2, A549, and PC3, as the model tumor cell lines, has been carried out before with concentrations ranging from 0.75 to 50 µg/mL. While HMWC showed significantly higher cytotoxicity toward human HepG2 and A549 than PC3 cells, in concordance with our study ,those results demonstrated that HMWC has antitumor activities against various tumor cell lines in vitro and might be applicable as an antitumor agent based on the biodegradability and biocompatibility of HMWC(32). Galbiati and et al reported the chitosan–folate microcapsules loaded with camptothecin significantly reduced the proliferation of HeLa tumor cells, while they have a negligible effect on fibroblasts (33). 

 In conclusion, two types of chitosan with different molecular weights and DDA are implicated to exhibit growth inhibitory effects against HeLa, Saos-2 and MCF-7 as tumor cell lines. It might be an option for treatment of cancer with fewer side effects and more studies involving *in vivo *experiments, drug formulation and clinical trials would be necessary in the future.
